# Neural-Dural Transition at the Thoracic and Lumbar Spinal Nerve Roots: A Histological Study of Human Late-Stage Fetuses

**DOI:** 10.1155/2016/8163519

**Published:** 2016-03-16

**Authors:** Kwang Ho Cho, Zhe Wu Jin, Hiroshi Abe, Shunichi Shibata, Gen Murakami, Jose Francisco Rodríguez-Vázquez

**Affiliations:** ^1^Department of Neurology, Wonkwang University School of Medicine and Hospital, Institute of Wonkwang Medical Science, Iksan 570-711, Republic of Korea; ^2^Department of Anatomy, Histology and Embryology, Yanbian University Medical College, Yanji, Jilin, China; ^3^Department of Anatomy, Akita University School of Medicine, Akita 010-8502, Japan; ^4^Maxillofacial Anatomy, Department of Maxillofacial Biology, Tokyo Medical and Dental University Graduate School, Tokyo, Japan; ^5^Division of Internal Medicine, Iwamizawa Kojin-kai Hospital, Iwamizawa 068-0833, Japan; ^6^Department of Anatomy and Human Embryology, Institute of Embryology, Faculty of Medicine, Complutense University, Madrid, Spain

## Abstract

Epidural blocks have been used extensively in infants. However, little histological information is available on the immature neural-dural transition. The neural-dural transition was histologically investigated in 12 late-stage (28–30 weeks) fetuses. The dural sheath of the spinal cord was observed to always continue along the nerve roots with varying thicknesses between specimens and segments, while the dorsal root ganglion sheath was usually very thin or unclear. Immature neural-dural transitions were associated with effective anesthesia. The posterior radicular artery was near the dorsal root ganglion and/or embedded in the nerve root, whereas the anterior radicular artery was separated from the nearest nerve root. The anterior radicular artery was not associated with the dural sheath but with thin mesenchymal tissue. The numbers of radicular arteries tended to become smaller in larger specimens. Likewise, larger specimens of the upper thoracic and lower lumbar segments did not show the artery. Therefore, elimination of the radicular arteries to form a single artery of Adamkiewicz was occurring in late-stage fetuses. The epidural space was filled with veins, and the loose tissue space extended ventrolaterally to the subpleural tissue between the ribs. Consequently, epidural blocks in infants require special attention although immature neural-dural transitions seemed to increase the effect.

## 1. Introduction

Epidural blocks have been used extensively in infants [1], with transesophageal echocardiography providing good guidance for anesthesiologists [2]. The immature and loose tissues of infants should facilitate the spread of injectate through the epidural and paravertebral spaces. However, no histological information is available on the neural-dural transition, especially in infants. To the best of our knowledge, the human fetus materials examined in most previous studies of the spinal dura [3, 4] were from the embryonic and early fetal periods and not the late fetal stages as those studies focused on the origin and differentiation of these structures and not their topographical anatomy. Thus, the clinical relevance of those studies for pediatric anesthesia is unclear.

Rodionov and Asfandiyarov [5] conducted an excellent study of the epidural space in fetuses younger than 12 weeks. Limited histological information was provided about older fetuses (15–30 weeks) by Jin et al. [6] who showed that the basic configuration of the fetal dura is comprised of the periosteal and meningeal layers, which is similar to that in adults. However, their focus was not on the neural-dural transition but on ossification in the posterior longitudinal ligaments. Likewise, the study of Munkácsi [7] also had a different focus, showing that most parts of the posterior, lateral, and anterior ligaments (intermediate layers of the spinal leptomeninges, Williams [8]) were resorbed during fetal development. Therefore, in order to elucidate basic anatomical information in order to allow for the conductance of safe and reasonable anesthesia and surgery of the infant spinal cord, we aimed to examine the structures surrounding the nerve roots in histological sections of late-stage fetuses.

The greater radicular artery of Adamkiewicz has interesting features in adults. It does not usually originate from the thicker intercostal/lumbar artery [9], and its arterial wall is specifically elastic [10]. Logically, in the early stage of fetal development, a pair of anterior and posterior radicular arteries should penetrate the dural sheath to enter the subarachnoid space at every segment of the spinal nerves. However, in the later stage, a few of the anterior arteries are likely to maintain the growth, whereas most of the arteries, especially the posterior ones, should be eliminated. Alternatively, their supply area becomes limited to the dorsal root ganglion (DRG). In addition, the arterial-dural transition is likely to change during the elimination process. Therefore, another aim of this study was to examine how the excess numbers of radicular arteries are eliminated in order to form the greater radicular artery. Using a resin-injection method, Undi et al. [11] and Zawiliński et al. [12] described the details of the fetal vascular anatomy in the spinal cord, and they summarized the remodeling of the radicular arteries as “a decrease in their number and transition from regular to irregular distribution.” However, due to the corrosion that occurs when making the resin cast clear, the dura and nerve were all removed. Consequently, the second aim of this study was to describe the arterial-dural transition during the elimination process.

## 2. Materials and Methods

This study was performed in accordance with the provisions of the Declaration of Helsinki of 1995 (as revised in 2000 in Edinburgh). We examined semiserial paraffin sections (0.5 mm interval, 10 *μ*m thickness) of the vertebral columns from 12 human fetuses (28–30 weeks; crown-rump length (CRL), 230–260 mm). These specimens included 18-19 pairs of spinal nerve roots and dorsal root ganglia that were from the first thoracic to the first or second sacral levels as well as the most proximal part of all 12 ribs. After removal of the posterior parts of the neural arch, including the spinous processes and the associated back muscles and ligaments, the specimens were decalcified by incubating them at room temperature in Plank-Rychlo's solution (AlCl_2_/6H_2_O, 7.0 w/v%; HCl, 3.6; and HCOOH, 4.6) for 6-7 days. We used the routine paraffin procedure to prepare frontal sections or tilted frontal sections that were tilted anteriorly on the right side and posteriorly on the left side from the dorsal side to the vertebral body for histology. All of the sections were stained with hematoxylin and eosin. The specimens used in this study were part of the large collection maintained at the Institute of Embryology of Universidad Complutense Madrid and were products of miscarriages or ectopic pregnancies managed at the Department of Obstetrics at the university. The study protocol was approved by our university ethics committee (number B08/374).

## 3. Results

**Figure 1:**
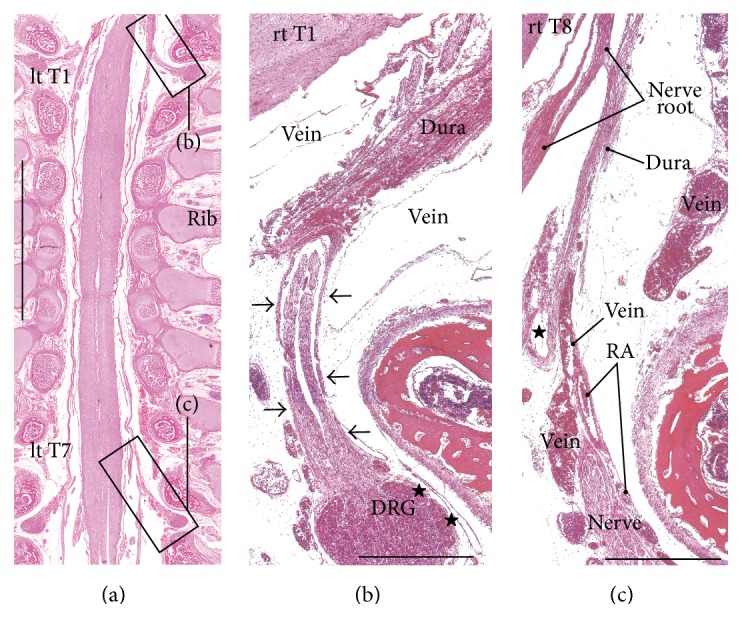
Thoracic nerve roots in a fetus with a crown-rump length (CRL) of 230 mm. (a) displays a posterior view of the thoracic spinal cord. In the right hand side of (a), a pleural space is seen between the ribs. (b), which corresponds to the square in (a), exhibits a sheath (arrows) of the right first thoracic nerve (rt T1). The sheath protrudes from the meningeal layer of the dura (dura) and continues to a thinner sheath (stars) around the dorsal root ganglion (DRG). (c), which corresponds to the other square in (a), shows a radicular artery (RA) running outside of a sheath (star) of the right eighth thoracic nerve (rt T8). Scale bars: 10 mm in (a), 1 mm in (b) and (c).

**Figure 2:**
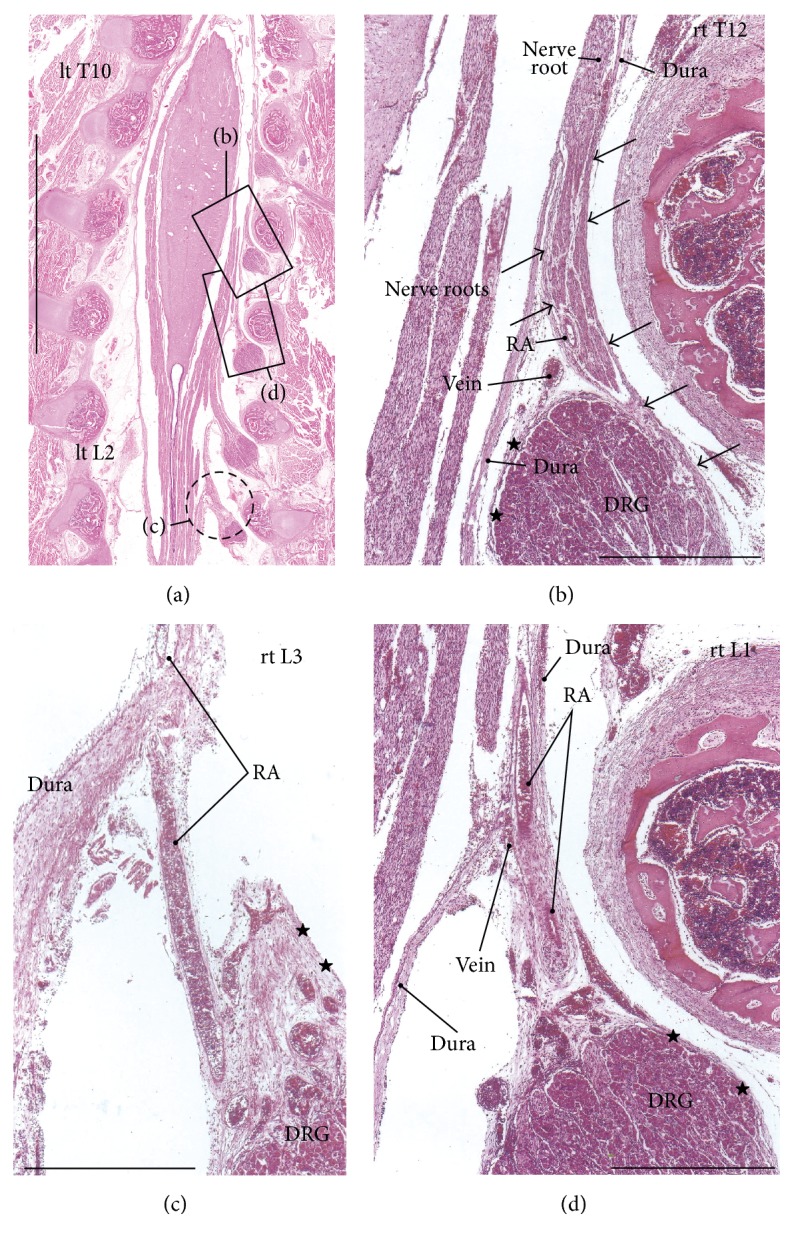
Thoracic and upper lumbar nerve roots in a fetus with a crown-rump length (CRL) of 230 mm. This is the same specimen that is shown in Figure 1. (a) displays a posterior view of the cord, including the conus medullaris. (b), which corresponds to a square in (a), shows a sheath (arrows) of the right twelfth thoracic nerve (rt T12). The sheath protrudes from the meningeal layer of the dura (dura) and continues to a sheath (stars) of the dorsal root ganglion (DRG). A radicular artery (RA) is embedded in the 12th nerve root. (c), which is a section that is 0.5 mm anterior to (a) (a circle by interrupted line), exhibits a radicular artery (RA) at the right third lumbar level (rt L3). A sheath of the DRG is cut tangentially (stars). (d), which corresponds to the other square in (a), exhibits RA running outside of the sheath of the right first lumbar nerve (rt L1). Scale bars: 10 mm in (a); 1 mm in (b)–(d).

**Figure 3:**
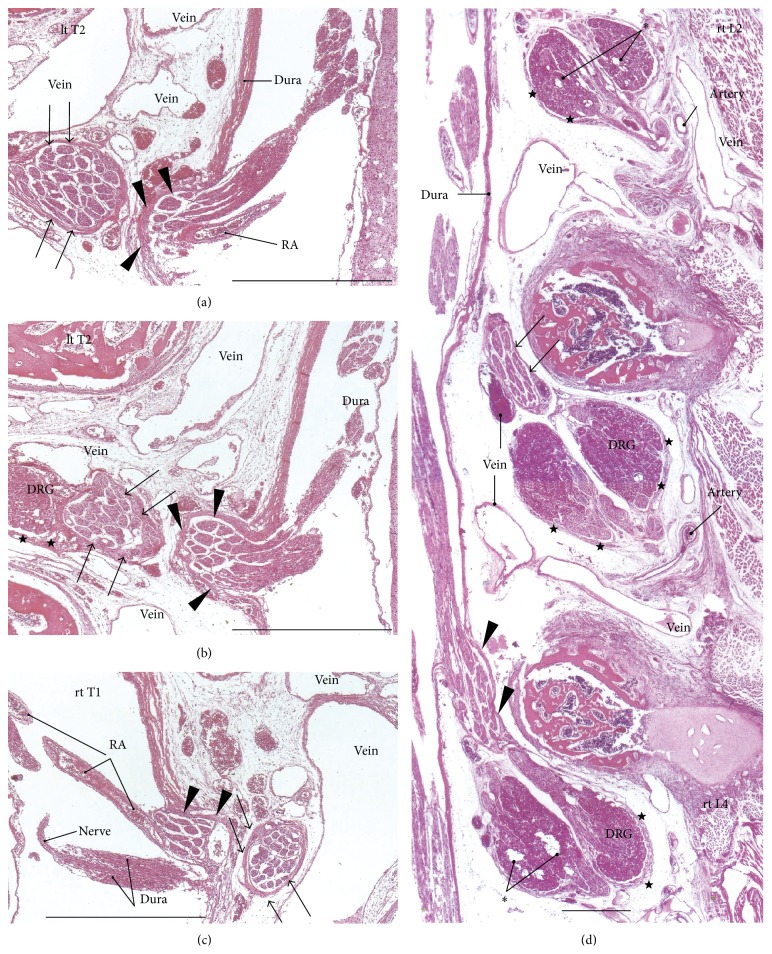
Thoracic and lumbar nerve roots in a fetus with a crown-rump length (CRL) of 235 mm. (a) and (b) (different sections with a 1-mm interval) display the left second thoracic nerve (lt T2) and its concomitant radicular artery (RA), which exhibited a pocket-like protrusion (arrowheads) of the meningeal layer of the dura (dura). A continuation of the dura (arrows) surrounds the nerve root and continues to a sheath (stars) of the dorsal root ganglion (DRG). (c) also exhibits the pocket-like protrusion (arrowheads) of the dura for the right first thoracic nerve (rt T1) and its concomitant RA. The dura continues to a nerve sheath (arrows). (d), including the right second–fourth lumbar nerves, shows a bilobule configuration of the dorsal nerve ganglia. The stars indicate the sheaths of the ganglion. The asterisks in (d) indicate tissue that was damaged during the histological procedure. All scale bars, 1 mm.

**Figure 4:**
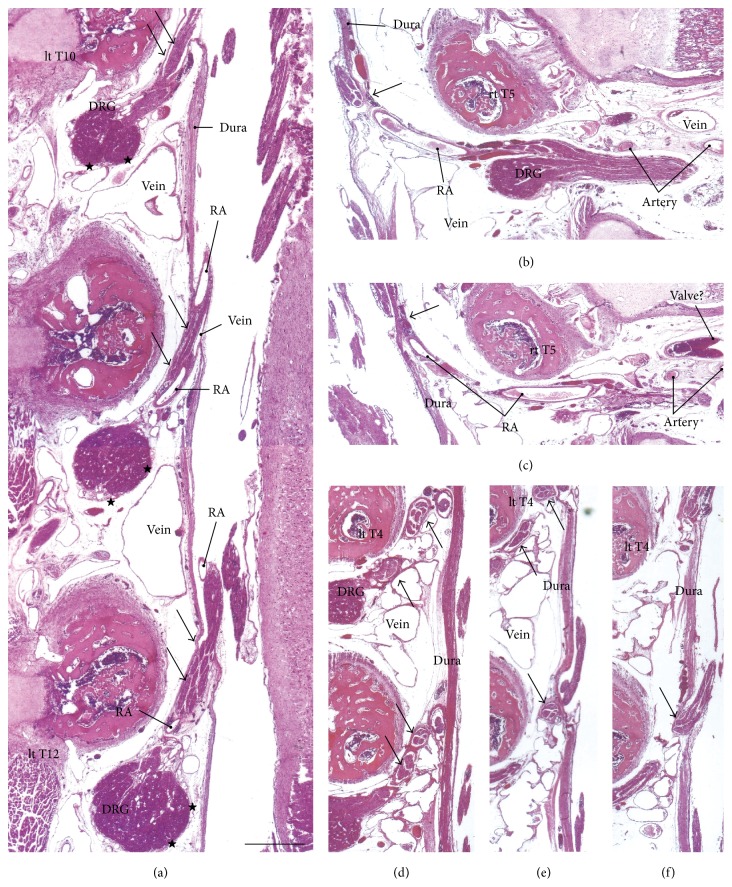
Thoracic and lumbar nerve roots in a fetus with a crown-rump length (CRL) of 245 mm. (a) The left tenth–twelfth thoracic nerves and a radicular artery (RA) and vein with a sheath (arrows) that is continuous with the meningeal layer of the dura (dura). The dorsal root ganglion (DRG) is covered by a thin sheath (stars). (b) and (c) (different sections with a 0.5-mm interval) display RA at the right fifth thoracic nerve level (rt T5): the artery appears not to accompany a protrusion (arrow) of the dura. (d)–(f) (different sections with a 0.5-mm interval) exhibit tortuous courses of the left fourth and fifth thoracic nerve roots. The nerves are surrounded by a continuation of the dura (arrows). All of the panels are shown at the same magnification (scale bar in (a), 1 mm).

**Figure 5:**
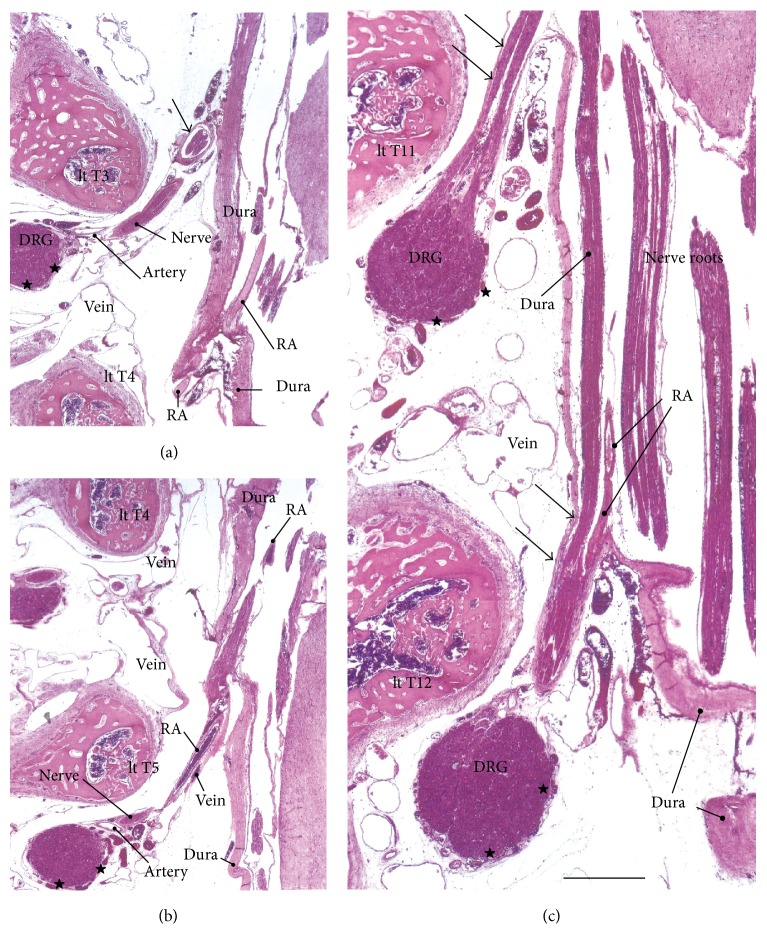
Thoracic and lumbar nerve roots in a fetus with a crown-rump length (CRL) of 250 mm. (a) and (b) (different sections with a 0.5-mm interval) display the left third thoracic nerve (lt T3) as well as two radicular arteries (RA) at the fourth and fifth thoracic nerve levels. (c) includes the left eleventh and twelfth thoracic nerves and a concomitant RA. In the three panels, the meningeal layer of the dura (dura) is thick, but the nerve sheath (arrow) is thin. A sheath of the dorsal root ganglion (DRG) is thin or unclear (stars). All of the panels are shown at the same magnification (scale bar in (c), 1 mm).

In the frontal and tilted frontal sections, the nerve root and its associated DRG were not usually seen in the series of continuous multiple segments but were seen in the 2-3 segments that were between them, which was possibly due to slight curvature of the vertebral column (Figures 1 and 2). In this study, we examined multiple sections in order to compare multiple segments. In order to clearly show the differences between specimens, we prepared one figure for each specimen, except for Figures 1 and 2. Thus, the figures show five of the 12 specimens that were examined. The inner or meningeal layer of the dura mater was thick (more than 0.1 mm). Below, the term* dural sheath* is used to refer to the inner layer of the dura as opposed to the term* periosteum*, which is used to refer to the outer layer of the dura. In addition, we examined the thin leptomeninges that were near but not attached to the spinal cord (Figures 3(a), 4(a), and 5(a)–5(c)) and that appeared to be arachnoid mater and not pia mater. Nevertheless, the leptomeninges did not attach to the dural sheath and were clearly separated from the dura by the intradural courses of the nerve roots and radicular arteries.

**Figure 6:**
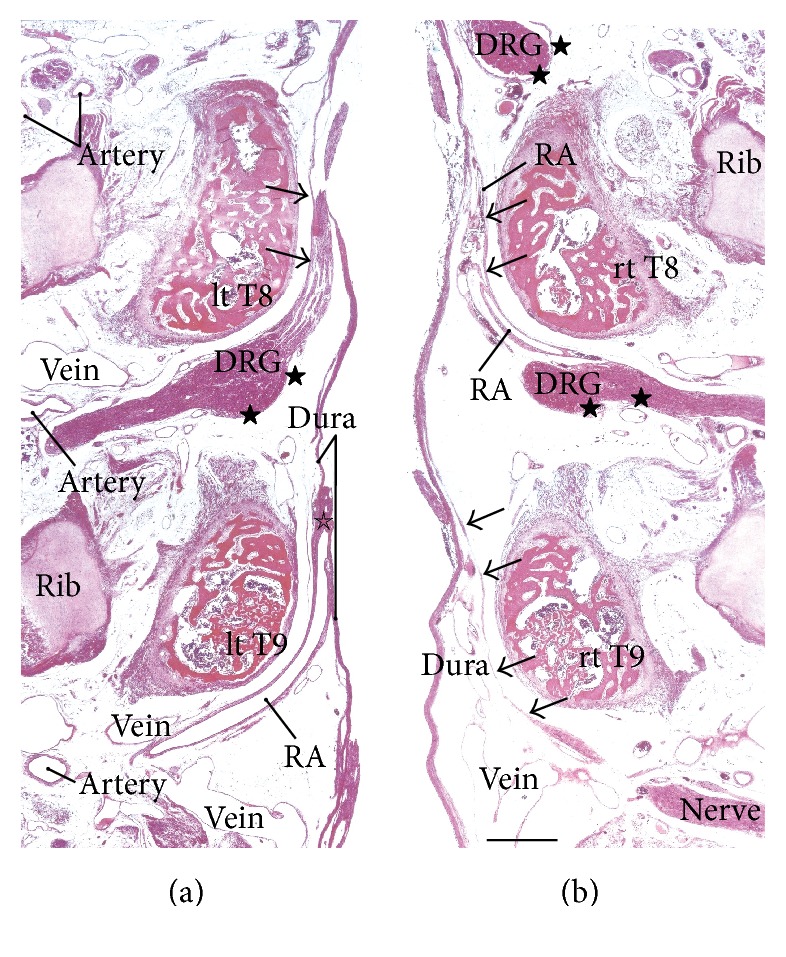
Thoracic and lumbar nerve roots in a fetus with a crown-rump length (CRL) of 260 mm. (a) and (b) display the bilateral eighth and ninth thoracic nerves (T8, T9) and concomitant radicular arteries (RA). The loose tissue space between the ribs continues to the subpleural space (not shown). The nerve sheath (arrows) is thin, and a sheath (stars) of the dorsal root ganglion (DRG) is unclear. The two panels are shown at the same magnification (scale bar in (b), 1 mm).

**Figure 7:**
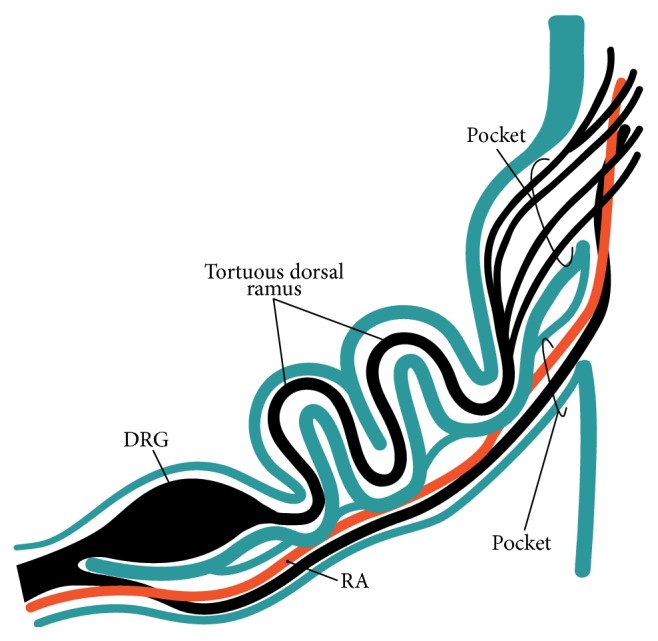
A diagram showing a tortuous course of the dorsal ramus starting from a large pocket-like protrusion of the dura. Note a difference in thickness of the dural sheath (blue) between the dorsal root ganglion (DRG) and the nerve rootlets from the spinal cord. In this diagram, the ventral ramus also accompanies a small pocket but it takes a straight course with the anterior radicular artery (red, RA).

The dural sheath of the spinal cord continued to the nerve roots in all of the specimens (Figures 1(b), 2(b), and 3–6), but the thickness along the nerve root varied considerably between specimens and between segments. Even if it was thicker than 0.1 mm, the sheath of the associated DRG was usually very thin or unclear (Figures 1(b), 2(b), 2(d), 3(d), 4(a), 5(a)–5(c), 6(a), and 6(b)). The DRG was bilobulated at the lumbar level in two of the 12 sections, and these two lobules merged together with the dorsal ramus (Figure 3(d)). A pocket-like protrusion of the dura along the nerve root was evident in half of the specimens (CRLs, 235 mm, 240 mm, 242 mm, 245 mm, 250 mm, and 252 mm; Figures 3–5). The pocket was likely to include both of the ventral and dorsal rami and when the nerve took a tortuous course, it had the appearance of a string of beads or a rosary (Figures 4(d)–4(f)). In the other half, the nerve root appeared to cross the dura obliquely (Figures 1(b), 2(b), and 6(a)). The pocket-like morphology of the dura as well as a tortuous course of the nerve root is schematically shown in Figure 7.

At all of the thoracic and lumbar segments, the dural sheath of the spinal cord faced the internal vertebral venous plexus that had a thin endothelium and other loose tissues. Because these tissues around the dura were very thick (almost 1 mm), we did not find any connections between the dural sheath and periosteum of the vertebral elements, even near and along the nerve root. The periosteum appeared not to form a peripheral nerve sheath. The loose tissues along the dural sheath extended laterally between the ribs and ended at the dorsomedial margins of the levator costae muscles (Figures 3(d) and 4(a)) and extended anterolaterally beneath the pleura (Figure 1(a)). Near the conus medullaris, the dural sheath was more or less folded, and it appeared as multiple protrusions that were irregularly shaped that contained sacral and lower lumbar nerve roots in some (Figure 5(c)). The internal venous plexus was well developed and the epidural space was filled with veins. DRG was usually surrounded by veins. In a part of the external venous plexus between ribs, valve-like structures were rarely seen (Figure 4(c)). Overall, the morphologies of the neural-dural transition were often symmetrical.

At all of the segments, the segmental arterial branch reached the DRG or a site near the DRG, and the nerve roots appeared to often lose their arterial supply. Despite their smaller thicknesses (0.1–0.3 mm) compared with the concomitant veins, the radicular arteries were discriminated from veins by their thick walls. Smooth muscles were not found along either arteries or veins in and along the dural sheath. The posterior artery tended to be near the DRG and/or embedded in the nerve root (Figures 2(b), 3(a), 3(c), 4(a), and 5(c)), whereas the anterior artery tended to be separate and distant from the nearest nerve root (Figures 2(c), 2(d), 4(a), 5(a), 5(b), 6(a), and 6(b)). Thus, the pocket-like protrusions of the dura did not contain the anterior artery (Figure 1(c)) but did contain the posterior artery (Figures 3(a) and 5(c)). Thin mesenchymal tissue, and not the anterior artery, was found with the continuation of the dural sheath (Figures 2(c), 5(b), 6(a), and 6(b)). The distributions of the radicular arteries are summarized in Table 1. The numbers of radicular arteries tended to decrease in larger specimens. Likewise, the arteries were not observed in the upper thoracic and lower lumbar segments in the larger specimens. In contrast to the morphologies of the neural-dural transitions, the presence of the radicular artery exhibited a left/right difference.

## 4. Discussion

The present study demonstrated the regional anatomy of the spinal column, especially the epidural space, of late-stage fetuses, and the anatomy appeared to be similar to the morphology of infants. Zawiliński et al. [12] used the resin cast method in three groups of human fetuses (10–14 weeks, 15–18 weeks, and 19–28 weeks) and reported segmental differences in the number of anterior and posterior radicoanastomotic arteries. They reported that (1) the numbers of both arteries did not differ between the second group (15–18 weeks) and third group (19–28 weeks) and (2) there was no anterior radicoanastomotic artery below the third lumbar segment. If we agree with the first point, the elimination of the radicular arteries likely starts at around 28 weeks. However, their resin casts might have demonstrated not only thick arteries but also very thin arteries. However, in contrast to their second point, we observed several arteries in the lower lumbar segment. The epidural space was filled with veins and DRG was also surrounded by veins. Thus, the present specimens appeared to show a stage later than the “developmental delay” in the posterior part of the fetal internal plexus [13]. We found valves in the external veins, but smooth muscles in the internal veins were not identified although these structures were recently discussed in relation to thermoregulation of the adult spinal cord [14, 15].

In late-stage fetuses as well as infants, spinal nerve roots should suffer from mechanical stress because of the great discrepancy in the growth rates between the spinal cord and the vertebral column. However, the rich venous plexus around the dural sheath might provide a buffer against the stress. The posterior radicular arteries were attached to or embedded in the corresponding nerve root. Therefore, the elimination of the radicular arteries in order to form the greater radicular artery of Adamkiewicz seemed to occur first in the posterior artery. In contrast, the anterior artery, which exhibited an intradural course that was independent of the nerve root, seemed to be rather free from the growth-related stress. However, the free intradural course of the anterior artery might also be weak. Accordingly, the level of the anterior artery would be narrowed and concentrated to a suitable vertebral level, such as the upper lumbar, which is seen in adults. In addition, the great radicular artery is seen on the left side in 65% of adults [8]. However, we did not find such laterality, which was possibly due to the limited number of specimens.

The pockets of the dural sheath were thick and exhibited a rosary-like arrangement that was possibly due to mismatch between the growth rates of the nerve roots and the maturation of the dura. The dural growth seemed to occur earlier, which made it hard for the nerve growth. Thus, the nerve pushed the dura, which resulted in a pocket, and the nerve exhibited a tortuous course in the periphery. Conversely, the dural pocket might have allowed the nerve root to adjust to the further growth of the vertebral column. The tortuous nerve course from the pocket-like protrusion of the dura was unlikely to allow the concomitant radicular artery since the latter requires a straight course (Figure 7). Notably, the morphologies of the neural-dural transitions showed significant individual differences instead of size-dependent differences. Therefore, the diffusion of epidural injectate into the nerve roots also appeared to have individual differences. Likewise, Jang et al. [16] have reported significant individual differences in the development of the filum terminale and the level of the conus medullaris in late-stage human fetuses. During the growth of the vertebral column, the mechanical stress that is exerted on the spinal nerve root might also vary between fetuses rather than between stages.

The spinal subdural space between the spinal dura and the arachnoid mater is the only potential space in normal adults as the arachnoid and dura are closely opposed. It does not connect with the subarachnoid space but continues for a short distance along the cranial and spinal nerves [8]. In the sections examined in this study, we always identified thin leptomeninges near but not attached to the spinal cord, and these leptomeninges appeared not to be pia mater but arachnoid mater. Nevertheless, the leptomeninges were not attached to the dural sheath but were clearly separated from the dura by the long intradural course of the nerve roots and radicular arteries. Perhaps, this arachnoid-like membrane corresponded to the lateral ligament that was described by Munkácsi [7] and the intermediate layer that was described by Williams [8].

Local anesthesia in adults is often applied by forcing the injectate to diffuse through the potential space, as is typically seen in C6 stellate ganglion blocks [17]. In infants, the loose tissues that surround the epidural space seem to make all routes of diffusion possible, even toward the subpleural space. Conversely, even local injections into deep back muscles might spread into the epidural space. However, the much greater extent of the internal vertebral venous plexus in infants compared to that in adults could affect the results so that it would be like an intravenous injection. The consistent and wide separation of the periosteum and dural sheath in the spinal canal could also result in an error by the anesthesiologist. Therefore, effective guidance is always necessary when attempting anesthesia in infants.

## Figures and Tables

**Table 1:** Distribution of thoracic and lumbar radicular arteries in 5 specimens shown in figures.

CRL	230 mm (Figures 1 and 2)	235 mm (Figure 3)	245 mm (Figure 4)	250 mm (Figure 5)	260 mm (Figure 6)
T1		R			
T2	R	L, R			
T3					
T4	L	L^*∗*^		L^*∗*^	
T5	L	L	R	L^*∗*^, R	
T6			R		
T7					
T8	R^*∗*^				R^*∗*^
T9		R^*∗*^		L	L^*∗*^
T10					
T11		R^*∗*^	L^*∗*^		R
T12	R		L	L	
L1	R	L^*∗*^		R	
L2	L^*∗*^, R^*∗*^				
L3	L^*∗*^, R				L
L4			R^*∗*^		
L5	L				
S1	L^*∗*^	L, R			

L, left; R, right.

^*∗*^Radicular artery is separated ventrally from a common nerve sheath surrounding the ventral and dorsal nerve roots at the corresponding levels.
